# The genetics of mitochondrial disease: dissecting mitochondrial pathology using multi‐omic pipelines

**DOI:** 10.1002/path.5641

**Published:** 2021-03-26

**Authors:** Charlotte L Alston, Sarah L Stenton, Gavin Hudson, Holger Prokisch, Robert W Taylor

**Affiliations:** ^1^ Wellcome Centre for Mitochondrial Research, Translational and Clinical Research Institute, Faculty of Medical Sciences Newcastle University Newcastle upon Tyne UK; ^2^ NHS Highly Specialised Services for Rare Mitochondrial Disorders, Royal Victoria Infirmary, Newcastle upon Tyne Hospitals NHS Foundation Trust Newcastle upon Tyne UK; ^3^ Institute of Human Genetics Technische Universität München München Germany; ^4^ Institute of Neurogenomics, Helmholtz Zentrum München Neuherberg Germany; ^5^ Wellcome Centre for Mitochondrial Research, Bioscience Institute, Faculty of Medical Sciences Newcastle University Newcastle upon Tyne UK

**Keywords:** mitochondrial pathology, genetic diagnosis, genomics, proteomics, metabolomics, mitochondrial disease

## Abstract

Mitochondria play essential roles in numerous metabolic pathways including the synthesis of adenosine triphosphate through oxidative phosphorylation. Clinically, mitochondrial diseases occur when there is mitochondrial dysfunction – manifesting at any age and affecting any organ system; tissues with high energy requirements, such as muscle and the brain, are often affected. The clinical heterogeneity is parallel to the degree of genetic heterogeneity associated with mitochondrial dysfunction. Around 10% of human genes are predicted to have a mitochondrial function, and defects in over 300 genes are reported to cause mitochondrial disease. Some involve the mitochondrial genome (mtDNA), but the vast majority occur within the nuclear genome. Except for a few specific genetic defects, there remains no cure for mitochondrial diseases, which means that a genetic diagnosis is imperative for genetic counselling and the provision of reproductive options for at‐risk families. Next‐generation sequencing strategies, particularly exome and whole‐genome sequencing, have revolutionised mitochondrial diagnostics such that the traditional muscle biopsy has largely been replaced with a minimally‐invasive blood sample for an unbiased approach to genetic diagnosis. Where these genomic approaches have not identified a causative defect, or where there is insufficient support for pathogenicity, additional functional investigations are required. The application of supplementary ‘omics’ technologies, including transcriptomics, proteomics, and metabolomics, has the potential to greatly improve diagnostic strategies. This review aims to demonstrate that whilst a molecular diagnosis can be achieved for many cases through next‐generation sequencing of blood DNA, the use of patient tissues and an integrated, multidisciplinary multi‐omics approach is pivotal for the diagnosis of more challenging cases. Moreover, the analysis of clinically relevant tissues from affected individuals remains crucial for understanding the molecular mechanisms underlying mitochondrial pathology. © 2021 The Authors. *The Journal of Pathology* published by John Wiley & Sons, Ltd. on behalf of The Pathological Society of Great Britain and Ireland.

## Introduction

Mitochondria are dynamic, intracellular organelles that mediate several key cellular functions, including apoptosis and calcium signalling, but importantly provide the primary source of cellular energy in the form of adenosine triphosphate (ATP) through oxidative phosphorylation (OXPHOS) [1]. The OXPHOS system comprises five multimeric complexes: NADH:ubiquinone oxidoreductase (complex I), succinate:ubiquinone oxidoreductase (complex II), ubiquinol:cytochrome *c* oxidoreductase (complex III), cytochrome *c* oxidase (complex IV), and the mitochondrial ATP synthase (complex V). Complexes I–IV represent the mitochondrial respiratory chain and coordinate, via mobile electron carriers, the pumping of protons across the inner mitochondrial membrane – from the mitochondrial matrix to the intermembrane space – to create an electrochemical gradient which is harnessed by complex V to synthesise ATP from ADP and inorganic phosphate. With the exception of complex II that is encoded entirely by autosomal genes, there is dual genetic control, and the viability of most eukaryotic cells is dependent upon highly co‐evolved interactions between nuclear DNA (nDNA) and the mitochondria's own polyploid genome (mtDNA), which collectively form the ‘mitonuclear genome’ [2]. The overwhelming majority of mitochondrial proteins are encoded within the nuclear genome (nDNA), although 13 polypeptides – and the 22 mt‐tRNA and 2 mt‐rRNAs genes required for their translation – are encoded by mtDNA. This genetic duality, with mitochondria dependent on ~1200 nDNA‐encoded proteins to support the majority of functions, combined with differing cellular demands and tissue‐specific expression of nuclear‐encoded mitochondrial proteins, presents unique challenges for understanding the role of mitochondria in disease. Mitochondrial disease is an umbrella term describing a group of heterogeneous genetic disorders that occur due to disrupted mitochondrial function. The ubiquity of mitochondria means that any organ or tissue can be affected, with those with the greatest energy requirement often most affected. Mitochondrial disease can manifest at any point in life, with clinical symptoms ranging from isolated symptoms, such as diabetes or exercise intolerance, to multi‐system involvement, such as Leigh syndrome (characterised by symmetrical lesions affecting the basal ganglia, thalamus, and brainstem, and associated with developmental regression) [3]; MELAS syndrome (the acronym derived from the hallmark features – Mitochondrial Encephalopathy, Lactic Acidosis and Stroke‐like episodes) [4]; Alpers syndrome, where the brain and liver are simultaneously affected [5]; or Pearson syndrome, characterised by sideroblastic anaemia and pancreatic dysfunction [6]. This clinical heterogeneity often makes the diagnosis of mitochondrial disease challenging and is further compounded by the vast genetic heterogeneity.

Unlike many genetic conditions, where the clinical phenotype is often caused by variation in a small number of candidate genes, around 10% of human genes are predicted to encode a mitochondrial protein. The latest build of the MitoCarta database (MitoCarta3.0 [7]; https://www.broadinstitute.org/mitocarta/mitocarta30-inventory-mammalian-mitochondrial-proteins-and-pathways) includes 1136 genes whose proteins are targeted to the mitochondrion and therefore represent 1136 a priori candidate genes for mitochondrial disease.

The clinical and genetic heterogeneity means that an unbiased, gene agnostic next‐generation sequencing strategy is becoming the most effective method for molecular diagnosis. For some patients, uncovering the underlying genetic defect remains challenging – for example, those harbouring deep intronic variants or variants involving proteins of unknown function – and these cases warrant additional avenues of investigation, particularly utilising transcriptomic or proteomic assessments, to firmly establish their diagnosis. Whilst there is no cure for mitochondrial disease, a genetic diagnosis is crucial for the provision of genetic counselling, patient management, and prognosis, whilst contributing to our understanding of the natural history of mitochondrial pathology.

This review discusses current diagnostic strategies for mitochondrial disease patients, which have evolved significantly over the last 10 years [8, 9]. As the diagnostic process moves away from invasive muscle biopsy in the first tier of investigation, this review aims to demonstrate that whilst a molecular diagnosis can be achieved for many cases through next‐generation sequencing using a blood DNA sample, the utility of patient tissues (particularly muscle and skin biopsy) and a multidisciplinary, multi‐omic approach are pivotal for the diagnosis of more challenging cases. Analysis of clinically relevant tissues from affected individuals remains a robust strategy for understanding the molecular mechanisms underlying mitochondrial pathology.

## Mitochondrial genomics

### Next‐generation sequencing (NGS) in the diagnosis of mitochondrial disease

**Figure 1:**
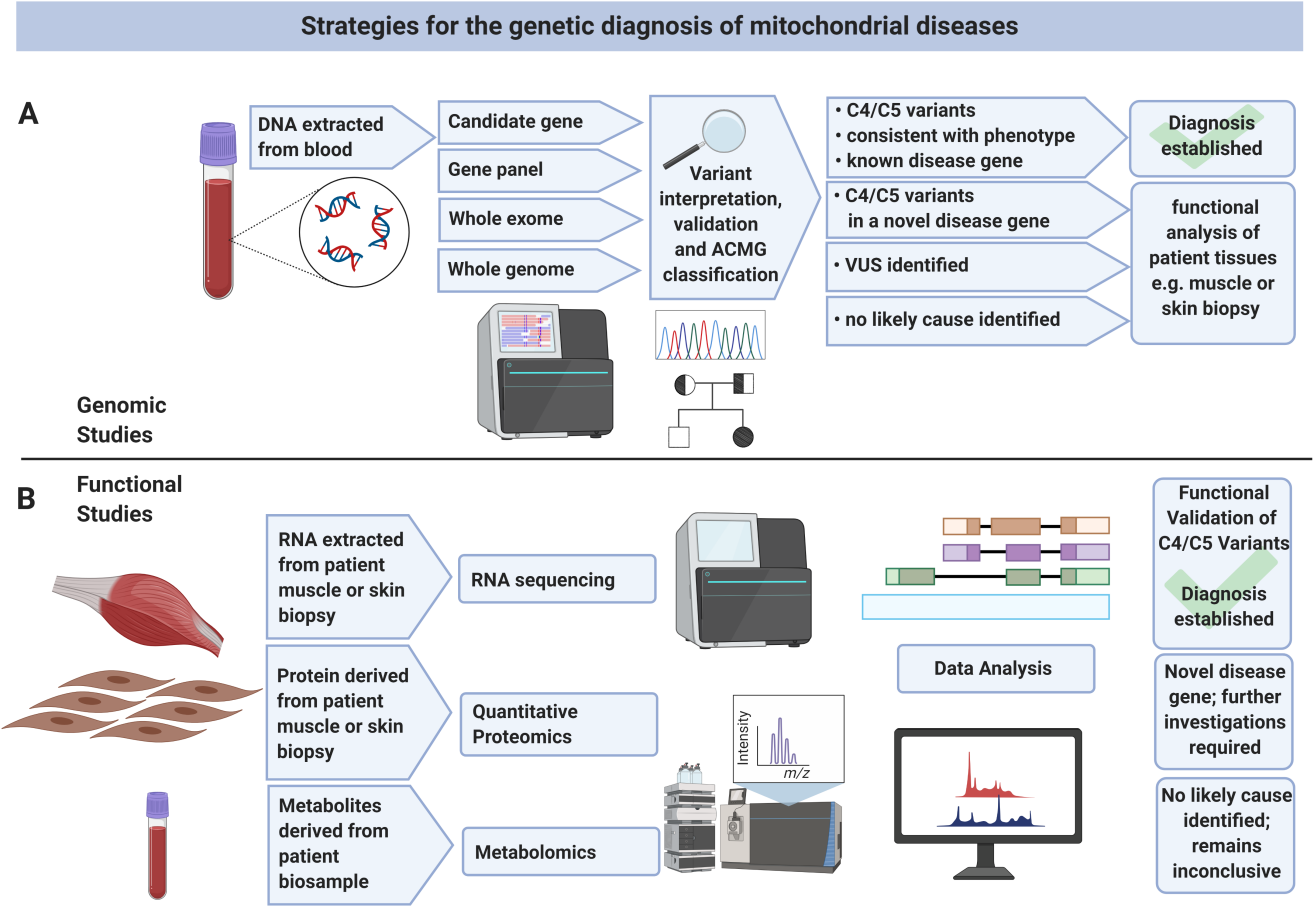
Strategies for the diagnosis of mitochondrial disease. (A) Illustration of a genomics pipeline, where genetic analysis of a blood‐derived DNA sample can be subject to either candidate gene, panel‐based sequencing, or unbiased WES/WGS. Genetic variants are assessed according to ACMG guidelines to determine those predicted to be causative variants (class 4 and class 5, being likely pathogenic and pathogenic, respectively); where these involve a known disease gene, they require no further analysis. For all other cases, including when likely/pathogenic variants are identified in genes not yet associated with mitochondrial disease, and variants of uncertain clinical significance (VUS; class 3), further functional analysis is required to confirm (or exclude) pathogenicity. (B) Depiction of the various ‘omics’ strategies, where patient biosamples (typically skeletal muscle or skin biopsy) are used for either proteomic, metabolomic or transcriptomic analysis. Data analysis reveals potential targets for genetic analysis or can either confirm or exclude pathogenicity of candidate variants obtained following genomic analysis. VUS: variant of uncertain pathological significance/class 3; C4: class 4/likely pathogenic and C5: class 5/pathogenic, according to American College of Medical Genetics (ACMG) guidelines for variant interpretation [10]. This figure was created with BioRender.com.

Genomic strategies within the diagnostic laboratory can be employed on a targeted or unbiased basis (Figure 1A). Variants are classified according to standardised American College of Medical Genetics (ACMG) guidelines [10], which utilise data combined from multiple sources including population frequency data, *in silico* predictions of consequence, and functional studies. Variant pathogenicity is defined as class 5 (pathogenic) or class 4 (likely pathogenic); conversely, neutrality is associated with class 2 (likely benign) and class 1 (benign). Variants with insufficient support for either pathogenicity or neutrality are ascribed class 3 status, representing a variant of uncertain significance (VUS). For patients in whom variant interpretation has prioritised one VUS (or more), further work is required to establish a diagnosis and functional analyses can be performed using a range of tissues or cell lines (Figure 1B); indeed, a multi‐omic approach is often the most effective strategy, as discussed in subsequent sections of this review.

Targeted approaches are typically most successful when there is a specific clinical phenotype, or where analysis of available biopsy material is suggestive of a distinct biochemical defect. For example, the observation of isolated mitochondrial complex I deficiency may prompt the analysis of a specific subset of candidate genes before embarking on an unbiased sequencing strategy [11, 12]. Other indicators which might support adopting a candidate gene strategy include a succinate peak on magnetic resonance spectroscopy (MRS), a hallmark of complex II deficiency and suggestive of *SDHx* gene involvement [13] or a maternal family history, suggestive of mtDNA involvement warranting specific mtDNA screening before nuclear gene analysis [14]. Similarly, isolated mitochondrial complex I deficiency would suggest a candidate gene pool of approximately 65 genes across both nDNA and mtDNA that encode either a structural subunit of complex I or a protein essential for its biogenesis [15, 16, 17, 18]. A targeted assessment of these genes yields a genetic diagnosis in approximately 50% of affected individuals identified with an isolated complex I deficiency [19], suggesting that there remains significant involvement from genes beyond the anticipated candidate gene pool.

The application of an unbiased approach to genetic diagnosis, such as whole‐exome (WES) or whole‐genome sequencing (WGS), should address the issue of an a priori candidate gene assumption and the clinical heterogeneity of mitochondrial disease; however, studies that have applied these techniques seldom exceed a diagnostic yield of 60%, even in well‐characterised patient cohorts [20, 21, 22, 23, 24, 25]. These genomic strategies cast a wide net, but the cumbersome data analysis historically resulted in lengthy diagnostic turnaround times. Conversely, a key advantage of targeted panel NGS was the delivery of a rapid diagnosis, with many diagnostic laboratories performing the technique and subsequent analyses locally.

More recently, improvements in laboratory workflows, together with the streamlining and unification of bioinformatic practices, have reduced the turnaround times for clinical exomes and unbiased WES or WGS. These have therefore emerged as an effective diagnostic option, even for cases demanding a rapid turnaround [26]. A recent study sequenced a custom panel of 1870 genes associated with early‐onset metabolic, neurological or dysmorphic presentations to critically ill children and their parents, achieving a diagnostic yield of 40%, with an average turnaround time of 7.5 days. Whilst these cases were not specifically enriched for suspected ‘mitochondrial disease’ cases, the fact that almost half of the diagnoses (5/13 diagnoses) involved mitochondrial proteins supports its utility for this patient group [27].

Indeed, so‐called ‘rapid genome sequencing’ (rGS) studies are emerging that improve on these already impressive turnaround times; the record for a diagnosis using whole rGS stands at just 19.5 h from sample receipt to the diagnosis being reported. The application of rapid exome or genome sequencing to critically ill neonates is particularly attractive as it requires only a blood sample and therefore negates the requirement for muscle biopsy and the general anaesthetic to facilitate its retrieval. A recent study revealed that 52/98 (53%) of the participating critically ill infants and children received a genetic diagnosis, of which 10% (*n* = 10) involved genes represented on MitoCarta [25]. When focusing on the metabolic cases, 7 of 8 cases had defects in MitoCarta genes.

Similarly, WES analysis of a cohort of children originating from South Asia [28], enriched with a clinical diagnosis of mitochondrial disease, resulted in a diagnosis rate of 35%. Interestingly, only half of the diagnoses involved MitoCarta3.0 genes. The vast majority of cases were associated with autosomal recessive inheritance, with the remaining patients harbouring a *de novo* pathogenic variant. This information is crucial for the provision of genetic counselling and the accurate estimation of recurrence risk for future pregnancies. Together, these studies validate the importance of an unbiased genetics‐first approach for these families in their time of clinical need [29, 30].

Even though WGS and WES using blood DNA are gaining some support as first‐tier tests in the diagnosis of mitochondrial disease [29], there remain a considerable proportion of cases where a causative variant eludes detection, either due to technological shortcomings (e.g. intronic variant in exome sequencing [31, 32]) or due to a lack of understanding of the implicated pathology (e.g. defects in genes not yet associated with human disease [18, 33]).

**Figure 2:**
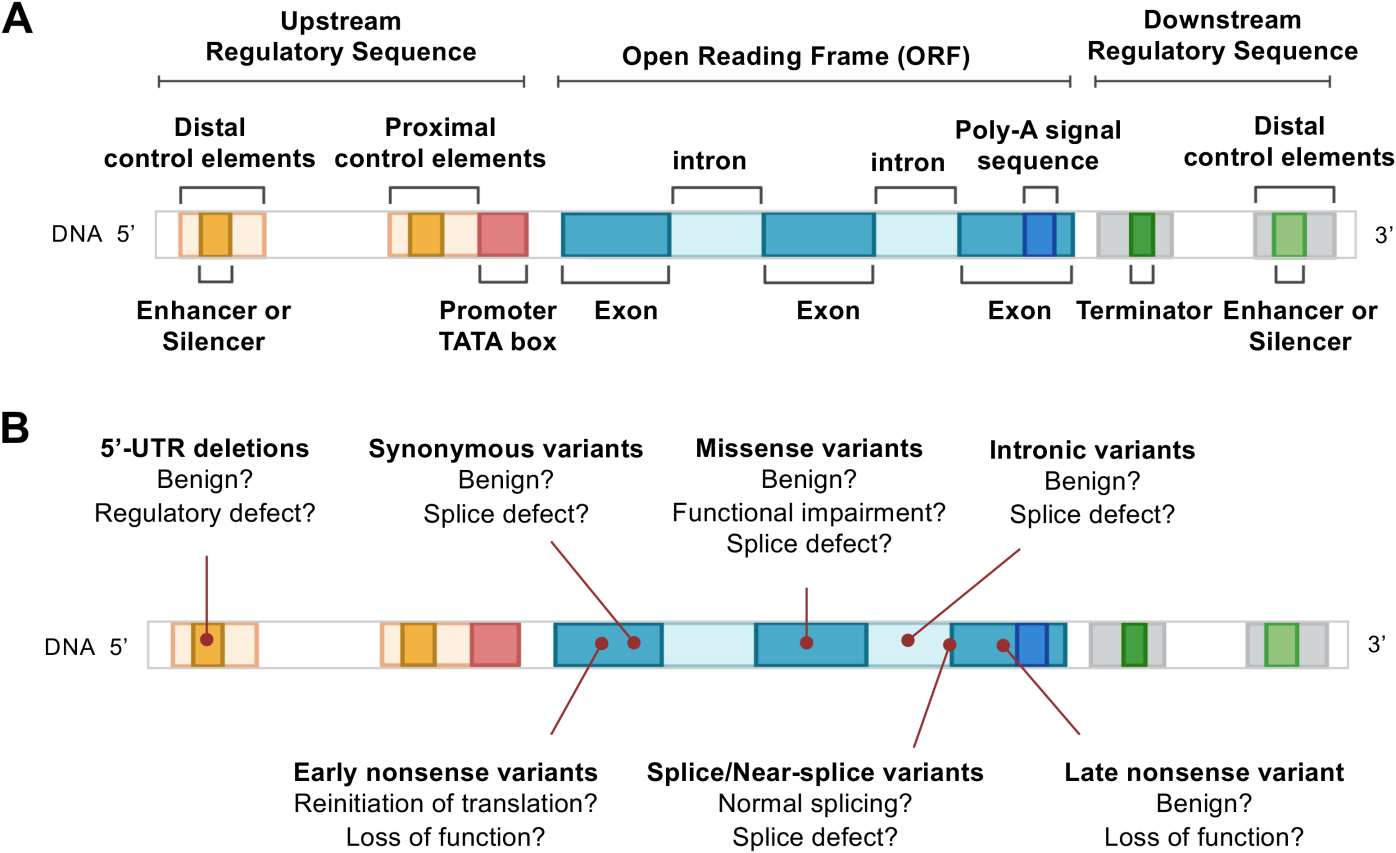
Overview of gene structure and examples demonstrating the utility of a multi‐omic approach to analysis. (A) Pictogram depiction of eukaryotic gene structure, with coding exons interspersed with intronic sequence representing the open reading frame (ORF) with 5'‐ and 3'‐untranslated regions (UTRs) flanking each gene. (B) Schematic illustrating the various types of VUS (or indeed presumed pathogenic/benign variants) and the possible outcomes following functional investigation using ‘omics’ approaches.

A graphical schematic of eukaryotic gene structure is provided in Figure 2A, whilst an overview of the current challenges in variant interpretation discussed in this review is illustrated in Figure 2B. Indeed, these challenges are not specific to the diagnosis of mitochondrial disease, but also apply to other heterogeneous conditions [34]. In addition to pathogenic variants within the coding region, pathogenicity can result from variants outside the coding region – either involving impaired mRNA transcription if they occur upstream or downstream of the gene's open reading frame, or by affecting mRNA splicing, where intronic variants activate or silence cryptic splice sites, thereby altering the mRNA sequence. Additionally, the anticipated functional consequence does not always correlate with the *in vivo* effect; for example, a missense variant may exert a pathogenic effect due to activation of a cryptic splice site, particularly if it occurs in the proximity of a natural splicing junction. Moreover, pathogenicity cannot always be assumed with transcripts containing premature stop codons – particularly those located within the 3'‐terminal exon – occasionally evading nonsense‐mediated decay. The vast majority of these defects require functional correlates to either confirm or exclude a pathogenic aetiology. It is hoped that the continual progression of our understanding of mitochondrial pathology will lead to a reduction in the pool of unsolved cases.

### Expansion of the molecular mechanisms underpinning mitochondrial diseases

WES and WGS strategies rely upon short‐read sequencing, with fragmented pieces of the genome aligned against the human genome reference sequence. Whilst short‐read exome sequencing is successful in establishing a genetic diagnosis for many individuals with mitochondrial disease, the remaining patients have an undetermined aetiology, harbouring, for example, genomic rearrangements or copy‐number variants (CNVs), deep intronic splicing variants, non‐coding variants or variants in an uncharacterised gene; whatever the underlying reason, the application of additional tests and techniques is required for their diagnosis. Arguably, whole‐genome sequencing has been demonstrated to perform reliable CNV calling and indeed deliver better coverage of the overall exome [35], with the accuracy of exome CNV analyses historically being regarded as suboptimal, often failing to detect small deletions. For this reason, CNV analyses are sometimes initiated only when a single likely pathogenic variant is identified in a strong candidate gene, although bioinformatics tools designed to reliably detect large, multi‐exon copy‐number variants are now incorporated into most pipelines [36]. Often the recognition of strong genotype–phenotype correlations can pave the way for the diagnosis of similar cases. For example, the established links between recurrent *de novo* deletions and duplications within the *ATAD3* gene cluster in cases of neonatal demise [37, 38] led to the investigation of *ATAD3* genes in paediatric mitochondrial disease, identifying pathogenic duplications, deletions, and missense variants involving *ATAD3* in mitochondrial disease patients [39, 40]. Generally, duplications involving mitochondrial genes remain a rare cause of mitochondrial disease, perhaps reflecting an ascertainment bias given that they may have been overlooked during sequencing analysis of WES data, which is further compounded by the difficulty in ascribing pathogenicity [41]; the advent of copy‐number variant analysis tools, such as ExomeDepth [42], and the application of whole‐genome sequencing within the diagnostic pathway will provide avenues to address this possibility. Long‐read sequencing technologies, such as those developed by Oxford Nanopore Technologies (ONT) and Pacific Biosciences (PacBio), deliver robust methodologies for the detection of large‐scale genomic structural variation. These have been successful in establishing the genetic diagnoses for some patients, including monozygotic twins presenting with myoclonic epilepsy in whom a *de novo CLN6* deletion was identified [43] using PacBio long read sequencing after exome sequencing failed to establish a diagnosis. Indeed, the application of ONT MinION long‐read sequencing was instrumental in characterising the duplication event within the *ATAD3* locus discussed previously [38].

Another rare mechanism that can underpin mitochondrial disease presentations is the uniparental inheritance of either segmental or entire chromosomes; the first cases of mitochondrial disease due to uniparental isodisomy (UPD) have been recently reported, involving paternal UPD of either chromosome 12 or chromosome 5 [44, 45]. The case involving chromosome 12 implicates two separate metabolic disorders, with paternal UPD giving rise to a homozygous pathogenic NM_025215.5:c.1122C>G, p.Tyr374* *PUS1* variant and a homozygous pathogenic NM_000289.5:c.237+1G>A *PFKM* splicing variant [44]. Similarly, paternal UPD of the entire chromosome 5 resulted in a homozygous NM_002495.4:c.350+5G>A *NDUFS4* splicing variant, involving a structural subunit of complex I [45]. Various pathogenic variants in *NDUFS4* have been reported, typically involving homozygous or compound heterozygous null alleles; therefore the presence of a biallelic splicing defect was consistent with the anticipated pathomechanism [19, 46, 47]. A normal genotype in the maternal DNA sample, however, supported a mechanism other than recessive inheritance, and UPD involving the entire chromosome 5 was confirmed. This case highlights the importance of considering UPD in the diagnosis of mitochondrial disease and whilst singleton analysis would fail to identify UPD, trio analysis or confirmation of carrier status for the parents is crucial given that UPD carries a low recurrence risk [48], which is important for genetic counselling.

## The functional characterisation of previously unreported mitochondrial disease genes

The functional characterisation of novel candidate genes is vital for the effective genetic diagnosis of patients with suspected mitochondrial disease – or indeed any other disease. The first mitochondrial disease gene uncovered by WES was *AARS2*, reported in 2011 [49]; pathogenic variants in 338 mitochondrial disease genes have since been reported in the literature [50]. Few would argue that the application of unbiased whole‐exome sequencing to undiagnosed cohorts by early adopters was the catalyst for the rapid expansion in mitochondrial candidate gene numbers.

Large‐scale sequencing projects have successfully revealed the degree of human genetic variation in the populations studied, but although these provide frequency data to facilitate identification of the ‘rare’ variants (those with an allele frequency less than 1% in the general population dataset), these rare variants still require further studies to determine which – if any – are causative [51, 52, 53]. A significant hurdle to the diagnostic success of genomic sequencing technologies, such as WES and WGS, is the interpretation of variants of uncertain significance (VUS) – predominantly missense, synonymous, and non‐coding variants – given the difficulty in confidently predicting their functional consequence. In recent years, multi‐omic assays such as transcriptomics (RNA‐seq) and quantitative proteomics have demonstrated value for the functional characterisation of such variants where clinically‐relevant patient material (e.g. muscle biopsy) is available. Moreover, RNA‐seq has been implemented as a high‐throughput discovery and validation tool in a diagnostic pipeline, negating the need for prioritisation of variants in individual genes prior to analysis [54, 55, 56, 57]. While proteomics has been utilised in individual cases [54, 58, 59, 60], it remains to be evaluated systematically.

Ascertaining the pathogenic consequence of genetic variants depends on successful capture of the gene product(s) by the assay. Fortunately, given the critical importance of mitochondrial function in the vast majority of cell types, mitochondrial disease genes are largely ubiquitously expressed [61]. Therefore, even if the disease only presents in an organ with high energy demands, perhaps one not easily amenable to biopsy such as the brain or heart, the functional consequences can be characterised using a more accessible tissue such as cultured fibroblasts or, to a lesser extent, whole blood. Using selected illustrative examples, we provide an overview of the diagnostic value of transcriptomic (RNA sequencing) and proteomic analyses in determining the genetic diagnosis for patients with suspected mitochondrial disease.

## Transcriptomics in suspected mitochondrial disease

**Figure 3:**
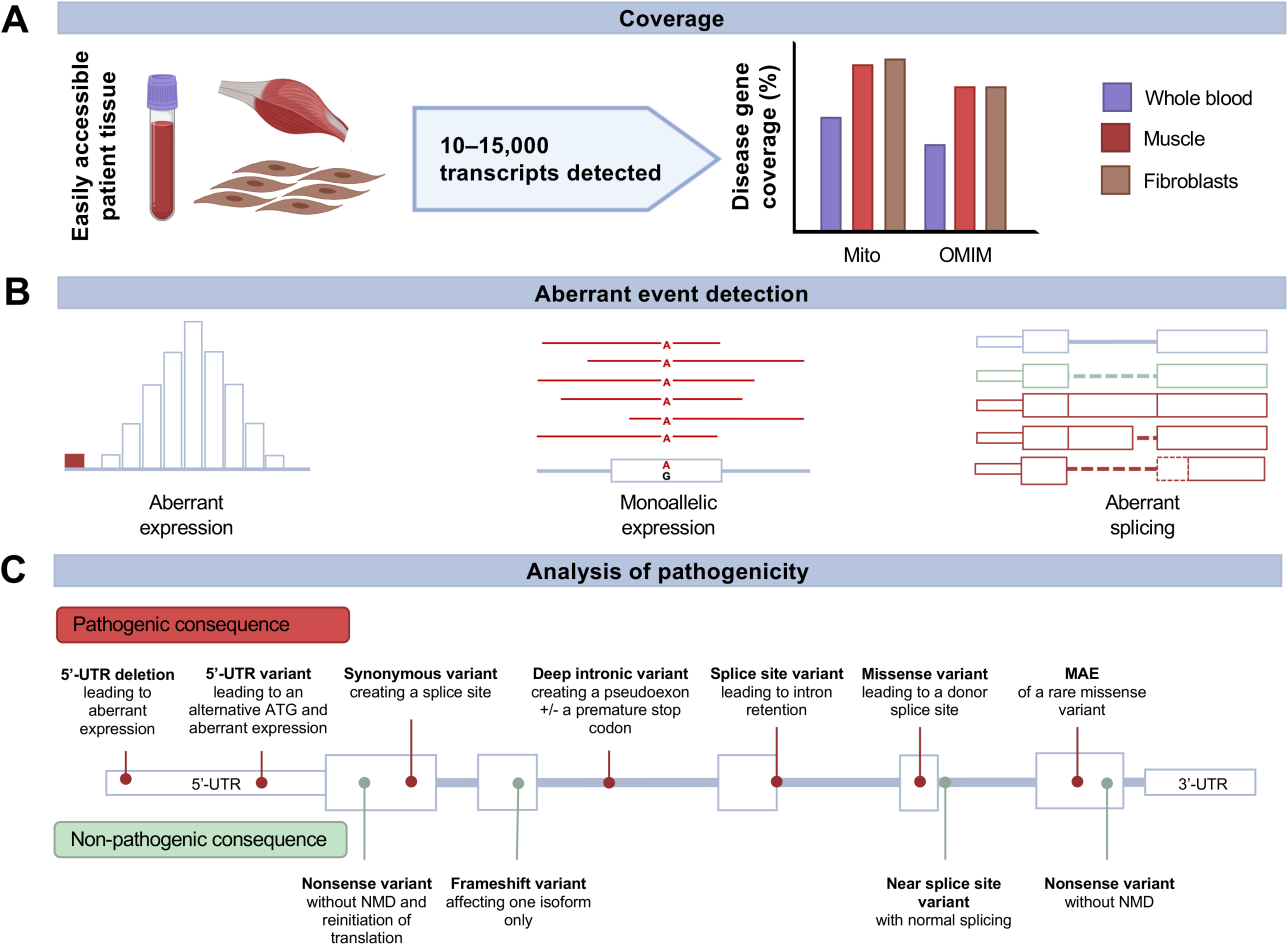
Transcriptomic analysis and evidence for variant pathogenicity in the diagnosis of mitochondrial disease. (A) Transcript detection and disease gene (mitochondrial and OMIM genes) coverage of RNA‐seq from easily accessible patient‐derived tissues. (B) Three different detection methods for aberrant expression events in RNA‐seq data. (C) Schematic demonstrating the various consequences that sequence variants can exert, both pathogenic and non‐pathogenic, on RNA abundance and isoforms. MAE, monoallelic expression; NMD, nonsense‐mediated decay; OMIM, Online Mendelian Inheritance in Man; UTR, untranslated region.

RNA sequencing (RNA‐seq) directly assesses transcript abundance and can provide valuable insight into the consequence of genetic variants of uncertain significance, particularly those situated near splice sites, located deep within introns and within regulatory (5′‐ and 3′‐UTR) regions. In clinically accessible tissues, 10–15 000 protein‐coding transcripts are detected [61, 62, 63] (Figure 3A). Analysis of the 338 known mitochondrial disease genes [50] reveals 60% to have transcripts detectable with an FPKM > 1 (Fragments Per Kilobase of transcript per Million mapped reads) in whole blood, whilst this figure rises to 90% and 86% in cultured fibroblasts and muscle, respectively. With regards to OMIM (Online Mendelian Inheritance in Man, https://www.ncbi.nlm.nih.gov/omim) disease genes, approximately 45% and 75% are expressed with an FPKM > 1, in blood and either fibroblasts or muscle, respectively (Figure 3A). Given that an estimated 10–60% of pathogenic variants are known to lead to abnormal splicing, RNA‐seq has the potential to capture the consequence of a substantial proportion of disease‐causing variation [64, 65, 66, 67, 68]. The application of RNA‐seq to a cohort of unsolved mitochondrial disease cases demonstrates its utility, resulting in a diagnostic yield of 15%; these patients had already undergone WES with no causative defects identified [54]. This diagnostic yield falls amongst those reported for other cohorts, ranging from 8% to 36% depending on the disease and tissue selection [54, 55, 56, 57]. In such studies, aberrant expression outliers, defined as an expression level outside of the expected physical range (such as |Z‐score| > 3), monoallelic expression of rare heterozygous variants, and aberrant splicing events were called to identify the genetic diagnosis (Figure 3B). In a recent study by the GTEx Consortium, only around 10% of such RNA‐seq outliers were found to be reproducible across tissues [69], of which monoallelic expression outliers were most consistently detectable [61, 69]. This discrepancy highlights the importance of appropriate tissue selection; resources (see web resources) including PAGE (Panel Analysis of Gene Expression) [57], GTEx (https://gtexportal.org) [61], and the Expression Atlas (https://www.ebi.ac.uk/gxa) [70] can be utilised to identify appropriate tissues expressing their disease genes of interest. Moreover, given the high variability in splicing across tissues, tissue‐specific splicing may also be assessed prior to tissue selection, such as by the tool MAJIQ‐CAT [71]. When the tissue of interest is not available or accessible, and coverage is suboptimal in available tissues, transdifferentiation from patient‐derived cultured fibroblasts has shown to be a valuable, although laborious, option [57]. There are many ways of detecting RNA‐seq outliers, and normalisation of the data is imperative; use of appropriate age‐ and sex‐matched controls [72, 73] and selection of the appropriate significance threshold are critical.

Focusing on mitochondrial disease genes, several variants with interesting pathogenic consequences detected by RNA‐seq have been reported in the literature (Figure 3C). For example, a rare hemizygous synonymous variant in *TAZ*: NM_000116.3:c.348C>T p.(Gly116Gly), a gene responsible for the mitochondrial membrane phospholipid and import machinery in which defects cause the X‐linked Barth syndrome, was shown to activate a cryptic donor sequence within exon 4, leading to the in‐frame deletion of eight amino acids (p.Lys117_Gly124del) within the tafazzin protein [74, 75]. As the variant was predicted to be synonymous, it was not prioritised during WES analysis. Reports of synonymous variants leading to splice site creation are increasing in the literature [55, 56, 57, 75]. Indeed, there are occurrences of missense variants that were subsequently found to exert a pathogenic effect due to aberrant mRNA splicing; an NM_024120.4:c.327G>C variant within *NDUFAF5* was identified by WES, predicting a p.Lys109Asn missense substitution, but additional analyses revealed that it in fact induces skipping of exon 3, on account of its location, immediately adjacent to the splicing donor consensus sequence [31]. A homozygous deletion, chr2:g.240964793_240964817del, within the 5'‐UTR region of *NDUFA10* that encodes a mitochondrial complex I subunit was initially identified in one patient [54] and later confirmed in an affected sibling, both of whom had an isolated complex I defect in skeletal muscle and a corresponding reduction in expression of the mRNA transcript [54]. A recurrent deep intronic variant (NM_016589.3:c.597‐1340A>G) within *TIMMDC1*, encoding a mitochondrial complex I assembly factor, was shown to activate a cryptic splice acceptor, leading to the creation of a pseudoexon and the introduction of a premature stop codon, p.(Gly199_Thr200ins5*). Upon reanalysis of the WGS data, the causative intronic variant was detectable within the pseudoexon, highlighting the utility of WGS over WES for identifying non‐coding variation. Additional support for pathogenicity was provided by further proteomic profiling, which revealed no residual TIMMDC1 protein in the patient [54]. Similar to *TIMMDC1*, a recent study described a recurrent deep intronic splicing variant in the mitochondrial complex I assembly factor *NDUFAF8* (NM_001086521.1:c.195+271C>T), resulting in loss of the predominant transcript and resultant protein [33]. A further study described a rare homozygous deep intronic variant within *NDUFB10*, a gene encoding a structural subunit of mitochondrial complex I [58]. The NM_004548.3:c.131‐442G>C variant was shown to activate a cryptic splicing acceptor resulting in exonisation of two stretches of intronic sequence, creating a premature stop codon in each case (p.Arg43fs*32 and p.Arg43fs*135, respectively). The consequence was reduced expression, corroborated by loss of the NDUFB10 subunit upon proteomic analysis; the possibility that the absence of NDUFB10 could be due to a technical issue, such as insufficient detection sensitivity, is eradicated by the concomitant decrease in the abundance of the other complex I subunits, specifically, with preservation of the subunits of the other complexes. This example highlights that evidence supporting pathogenicity may be apparent from the analysis of interacting partners or proteins involved in the same pathway [58]. In this case, the decreased levels of other complex I subunits acted as a proxy for NDUFB10, providing support for the pathogenicity of the *NDUFB10* variants. Such deep intronic variants are usually ignored by prediction tools [76], exemplified in *NDUFB10* by the *in silico* splice prediction algorithm SpliceAI [77], which showed a low confidence prediction that the variant would lead to an aberrant splicing event [58]. Moreover, multiple variants were detected in the region of the *NDUFB10* pseudoexon by WGS, demonstrating the importance of using a minigene splicing reporter assay to confirm the association of a variant of interest with the expected aberrant splicing event [58].

In addition to pseudoexon creation within the intron, pathogenic intron retention events are also emerging as a cause of Mendelian disease [57]. Many software tools have been designed to screen NGS data for intron retention events, although their robust detection evades most RNA‐seq analysis algorithms, which have focused on the detection of alternative splicing events only [78]. However, FRASER, a recently developed statistical method for RNA‐seq analysis [75], considers the number of non‐split reads including consensus splice sites to allow the detection of intron retention. The application of FRASER for reanalysis of RNA‐seq data led to the identification of intron retention in the *MCOLN1* gene, encoding the mucolipin 1 protein, thereby confirming the diagnosis of a lysosomal storage disorder: mucolipidosis type IV, in a suspected mitochondrial disease case that had been exhaustively investigated [54, 75]. Beyond variants described in mitochondrial disease genes, other interesting pathogenic variant consequences uncovered by RNA‐seq include missense variants leading to alternative splice sites and 5'‐UTR variants leading to the selection of an alternative initiation methionine [55].

RNA‐seq has proven useful in excluding the pathogenicity of prioritised VUS in suspected Mendelian disease patients (Figure 3C). This is exemplified by a homozygous NM_012123.3:c.1996C>T p.(Arg666*) variant in *MTO1*, a gene involved in mitochondrial RNA processing; the predicted homozygous nonsense variant was identified in a patient with an isolated mitochondrial complex III defect and was proven to be benign, given the absence of nonsense‐mediated mRNA decay and normal protein expression [79]. In other cases, direct and near‐splice site variants have been excluded due to evidence of normal splicing [55], or nonsense variants that evade nonsense‐mediated mRNA decay [54], or where there is evidence of translation re‐initiation downstream of the anticipated stop codon [80]. These cases suggest that the pathogenicity of a ‘loss‐of‐function’ variant, such as splice, nonsense, or frameshift variants, cannot be assumed to truly result in a loss of mRNA and resultant proteins without a functional correlate to provide supportive evidence. A more complex example is found in *FLAD1*, an essential gene involved in riboflavin metabolism and transport in which non‐lethal homozygous frameshift variants were identified (NM_025207.4:c.526_537delinsCA, p.Ala176Glnfs*8 and NM_025207.4:c.401_404del p.Phe134Cysfs*8) [81]. Both *FLAD1* variants affect a conserved domain upstream of the flavin adenine dinucleotide synthase (FADS) domain, and their identification, together with the unexpectedly preserved FADS activity, led to the discovery of an alternative functional RNA isoform [81]. This exemplifies a variant leading to a loss‐of‐function transcript isoform, which is partly complemented by an alternative unaffected isoform, thereby highlighting the importance of considering isoform‐specific pathology.

## Proteomics in suspected mitochondrial disease

**Figure 4:**
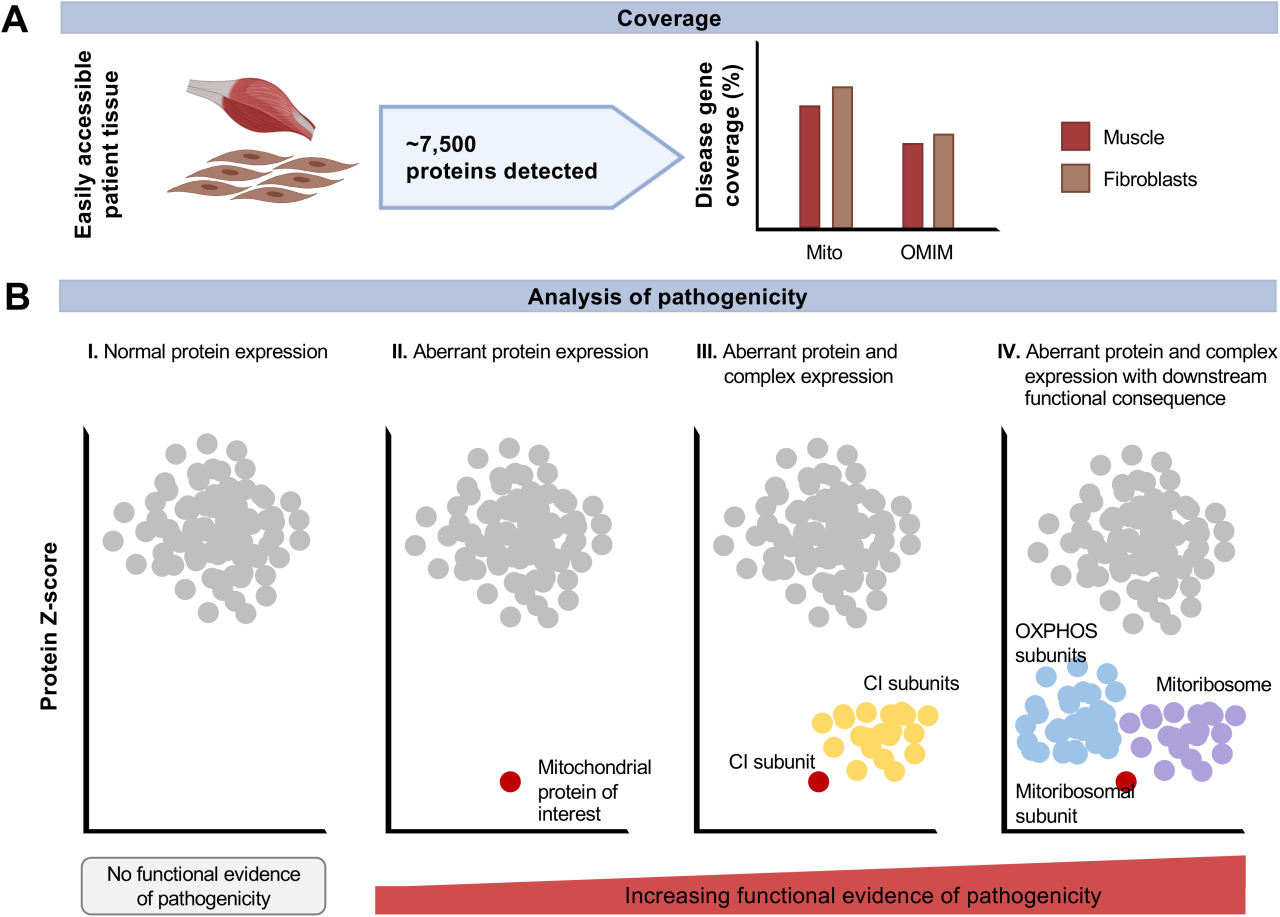
Proteomic evidence for variant pathogenicity in the diagnosis of mitochondrial disease. (A) Protein detection and disease gene coverage metrics associated with the proteomic analysis of easily accessible patient‐derived tissues. (B) Analysis of variant pathogenicity with illustrative examples: I. Variant(s) demonstrating protein expression within the physiological range (grey), either due to non‐pathogenicity or due to affecting protein function but not abundance. II. Variant(s) demonstrating pathogenicity by reduced protein expression (red) below the Z‐score threshold (representing protein fold‐change divided by the standard deviation of the normalised protein expression in the analysed population). III. Variant(s) demonstrating pathogenicity by reduced abundance of the protein of interest (red; for example, a complex I subunit) and interacting proteins (yellow, other complex I subunits). IV. Variant(s) demonstrating pathogenicity by reduced expression of the protein of interest (red; for example, a mitoribosomal subunit) and its complex (purple, other mitoribosomal subunits) with additional evidence of a downstream functional impairment (blue, OXPHOS subunits). CI: complex I; OMIM: Online Mendelian Inheritance in Man.

Proteomics enables accurate, increasingly sensitive, quantitative analysis of protein abundance and offers a functional snapshot of variant consequence. Akin to transcriptomic analyses, not only one but thousands of gene products can be studied simultaneously, allowing the application of one assay for the functional validation of many variants. The encoded proteins of approximately 65–75% of the mitochondrial disease genes and 40–50% of all OMIM disease genes can be quantified in muscle and fibroblasts, respectively [82] (Figure 4A). In contrast to RNA‐seq, proteomic analyses may allow investigation into the pathogenicity of missense variants, the most common class of pathogenic variant in mitochondrial disease and one of the most challenging in which to predict the associated functional consequence. Approximately 40% of missense variants are expected to cause reduced expression of the protein, due to resulting instability and subsequent degradation [83]. Though RNA‐seq has broader coverage, it does not predict the consequence at the protein level; proteomics arguably provides a better biological insight into the consequence of a defect, such as on the interaction partners, the respective complex, and the downstream function of the protein. In this regard, approximately 20% of mitochondrial and 45% of OMIM disease genes form part of a protein complex, according to the more than 2350 protein complexes listed in CORUM (http://mips.helmholtz-muenchen.de/corum/#download), and thereby a defect in one of these proteins may demonstrate a downstream consequence, providing an additional layer of supportive functional evidence for pathogenicity.

Here, we focus on interesting mitochondrial disease gene variants with pathogenic consequences detected by proteomic analyses (Figure 4B). Kremer *et al* utilised proteomics to evaluate the consequence of diminished RNA expression on protein level [54]. The study confirmed that the causative genetic defect may demonstrate reduced or diminished protein expression, as commonly investigated by western blotting for a protein of interest. Moreover, they found that loss of TIMMDC1, an assembly factor of mitochondrial complex I, led to the global reduction in the abundance of the assembled complex I holoenzyme. This exemplifies the power of proteomics to reveal the functional consequence of the defect on the interaction partners – here the structural subunits of mitochondrial complex I – and highlights the benefit of proteomics over established, but rate‐limiting, methodologies such as western blotting.

An overall mitochondrial complex I deficiency was similarly demonstrated by Helman *et al* as a result of the loss of the mitochondrial complex I subunit NDUFB10, attributed to presumed instability of the complex [58]. This supporting evidence of complex I deficiency was consistent with the previously described cellular model (*NDUFB10* knockout in HEK293T cells) [84]. To date, there are only two examples of the discovery of novel mitochondrial disease gene encoding proteins within a mitoribosomal subunit – *MRPS34* [59] and *PTCD3/MRPS39* [60] – through proteomic studies. In both examples, the defect led to the loss of the associated protein and a reduction of the entire small mitochondrial ribosomal subunit due to destabilisation of the complex. As anticipated, a reduction in OXPHOS subunit expression was observed, consistent with the mitoribosome being unable to translate mitochondrial mRNAs into their corresponding OXPHOS subunits. Interestingly, the subunit abundance was found to correlate closely with the cases' respiratory chain enzymology results, thereby validating the sensitivity of these assays through comparison to existing methods widely used in diagnostic laboratories. Such examples demonstrate the utility of proteomics in detecting the downstream functional consequence of the defect beyond its direct interaction partners.

Investigating protein interaction partners through proteomics also led to the identification of NDUFAF8 as the interaction partner of NDUFAF5 [18], a well‐characterised mitochondrial complex I assembly factor [85]. Akin to the deficiency in mitochondrial complex I subunits detected by proteomics in the TIMMDC1 defect, a global reduction in mitochondrial complex I subunits was detected following complexome profiling, a quantitative mass‐spectrometry assay described by Heide *et al* [86], in addition to a reduction in the assembled respirasome in the fibroblasts from a patient with bi‐allelic *NDUFAF8* defects [33]. Here, complexome analysis added further supporting information to the role of a novel mitochondrial disease gene in complex I assembly by combining blue native polyacrylamide gel electrophoresis (BN‐PAGE) with proteomics. Recently, this strategy has been effective in corroborating the pathogenicity of pathogenic variants involving other complex I subunits (NDUFC2 [87]) and assembly factors (TMEM126B [88, 89]), and in confirming the involvement of *TMEM70* in the assembly pathways of both complexes I and V [15]. Moreover, Edhager *et al* demonstrated the value of omics analyses in explaining phenotype variability [90]. Edhager *et al* employed a quantitative mitochondrial proteomic approach using fibroblast cell line pathogenic variants in *ACADS*, encoding a mitochondrial flavoprotein, to identify over 300 interacting proteins, and map protein perturbations to mitochondrial pathways in order to differentiate between mild and severe ACADS deficiency, in addition to confirming a severe reduction in the SCAD protein [90].

## Mitochondrial metabolomics

Metabolomic analysis has been instrumental in the diagnosis of certain mitochondrial diseases. Perhaps the most widely known examples are the treatable fatty acid oxidation disorders medium‐chain acyl‐coenzyme A dehydrogenase deficiency (MCADD) and very long‐chain acyl‐coenzyme A dehydrogenase deficiency (VLCADD) [91, 92], both typically rapidly assessed using sensitive and specific mass spectrometry‐based techniques as part of many global newborn screening programs [93, 94].

Whilst some metabolites such as lactate are non‐specific [95], metabolites identified following GC/MS urinary acid analysis can be suggestive of a particular metabolic pathway and candidate gene(s). A liquid chromatography quantitative time‐of‐flight (LC‐QTOF) mass spectrometry approach has been applied to effectively discriminate between various inborn errors of metabolism, using plasma from affected individuals [96]. This approach, in conjunction with candidate gene sequencing, could be useful in obtaining a genetic diagnosis for patients with certain complex mitochondrial aetiologies, without the requirement for muscle biopsy. Moreover, the association of particular biomarker profiles with particular gene defects could potentially result in a powerful tool to generate functional evidence to confirm (or indeed exclude) the pathogenicity of variants identified by whole‐exome or ‐genome sequencing. Whilst pathogenic variants in *PCCA* are a well‐established cause of propionyl‐CoA carboxylase deficiency, the pathogenicity of a novel homozygous NM_000282.2:c.1427G>C p.(Arg476Pro) *PCCA* missense variant was corroborated using a metabolomics approach. Tandem mass spectrometry (MS/MS) was performed on the patient's dried blood spots, revealing an elevated C3‐carnitine and C3‐/C2‐carnitine ratio, whilst urinary amino acids were evaluated using gas chromatography/mass spectrometry (GC/MS), which demonstrated increased excretion of methyl citrate and 3‐hydroxy‐propionate. The patient's metabolomic profiles, together with *in silico* modelling of the c.1427G>C p.(Arg476Pro) *PCCA* variant, facilitated its classification as ‘likely pathogenic’, thereby confirming the diagnosis [97].

Increased methacrylyl‐CoA and acryloyl‐CoA‐related metabolites were detected in a family with mitochondrial short‐chain enoyl‐CoA hydratase (SCEH) deficiency, which led to the analysis of the *ECHS1* gene [98]. Whole‐exome sequencing revealed no potential pathogenic variants within *ECHS1*, but a region of low read depth prompted further evaluation using Sanger sequencing, which revealed a homozygous pathogenic NM_004092.3:c.538A>G, p.(Thr180Ala) *ECHS1* variant, segregating across all three affected children. This example highlights the importance of confirmation by Sanger sequencing to validate NGS results and of a thorough interrogation of sequence data where there is strong clinical or biochemical support for a particular gene defect.

It is becoming clear that an integrated approach towards diagnosis is particularly powerful [99]; indeed, utilising proteomic, metabolomics, and genomic investigations led to the recent discovery of a novel cause of mitochondrial dysfunction involving *SHMT2*, which encodes a protein involved in 1‐carbon metabolism [100]. Recessively‐inherited pathogenic *SHMT2* variants were identified by WES in five children from four families who presented with a novel syndrome, with symptoms including dysmorphic features, intellectual disability, motor dysfunction (spastic paraparesis, ataxia, and/or peripheral neuropathy), ragged red fibres in muscle, cardiac abnormalities, and microcephaly. Extensive functional studies including modelling *hmt2* knockdown in *Drosophila* validate SHMT2 dysfunction as a cause of defective glycine metabolism [100]. Employing these multi‐omics approaches may also aid in our understanding of the phenotypic spectrum of individual diseases. This was exemplified by a case in the study by Kremer and co‐workers [54], where RNA‐seq, proteomics, and metabolomics provided complementary functional evidence of pathogenicity for a VUS in *ALDH18A1* in a patient where the phenotype did not overlap with the expected connective tissue disease [101]. Given the non‐overlapping phenotype, the diagnosis was not initially accepted by the referring clinician despite each ‘omic’ independently supporting pathogenicity. However, the phenotype has since been extended to include hereditary spastic paraplegia, cerebellar ataxia, and cognitive impairment [102, 103], supporting the initial omics finding. This may indicate that robust functionally derived evidence for VUS validation from independent omics is more reliable than the patient's phenotype, especially given the non‐specificity of the clinical, biochemical, and imaging phenotypes in mitochondrial disease.

## Concluding remarks

Overall, multi‐omics are increasingly replacing single gene product studies including qPCR, RT‐PCR, and western blotting. They are optimally used in a complementary fashion, as in single gene studies, to achieve the highest sensitivity and provide independent validation for each approach. Moreover, these assays can establish unexpected diagnoses and can provide much more information regarding the pathomechanism of the causative variant. The added diagnostic value of proteomic, transcriptomic, and metabolomics analyses not only encourages the routine collection of patient's biological samples, such as cultured fibroblasts from a minimally invasive skin biopsy, whole blood or diagnostic muscle biopsy but, importantly, increases our fundamental understanding of the underlying mitochondrial biology and pathology of mitochondrial disease.

## Author contributions statement

All the authors contributed to writing this review and to critical revision of the final version of the manuscript.
